# Defining objective clusters for rabies virus sequences using affinity propagation clustering

**DOI:** 10.1371/journal.pntd.0006182

**Published:** 2018-01-22

**Authors:** Susanne Fischer, Conrad M. Freuling, Thomas Müller, Florian Pfaff, Ulrich Bodenhofer, Dirk Höper, Mareike Fischer, Denise A. Marston, Anthony R. Fooks, Thomas C. Mettenleiter, Franz J. Conraths, Timo Homeier-Bachmann

**Affiliations:** 1 Friedrich-Loeffler-Institut, Federal Research Institute for Animal Health, Institute of Epidemiology, Greifswald-Insel Riems, Germany; 2 Friedrich-Loeffler-Institut, Federal Research Institute for Animal Health, Institute of Molecular Virology and Cell Biology, OIE Reference Laboratory for Rabies, WHO Collaborating Centre for Rabies Surveillance and Research, Greifswald-Insel Riems, Germany; 3 Friedrich-Loeffler-Institut, Federal Research Institute for Animal Health, Institute of Diagnostic Virology, Greifswald-Insel Riems, Germany; 4 Institute of Bioinformatics, Johannes Kepler University Linz, Linz, Austria; 5 Institute of Mathematics and Computer Science, University Greifswald, Greifswald, Germany; 6 Wildlife Zoonoses and Vector-Borne Diseases Research Group, Animal and Plant Health Agency (APHA), OIE Reference Laboratory for Rabies, WHO Collaborating Centre for Characterization of Lyssaviruses, Weybridge, United Kingdom; Wistar Institute, UNITED STATES

## Abstract

Rabies is caused by lyssaviruses, and is one of the oldest known zoonoses. In recent years, more than 21,000 nucleotide sequences of rabies viruses (RABV), from the prototype species rabies lyssavirus, have been deposited in public databases. Subsequent phylogenetic analyses in combination with metadata suggest geographic distributions of RABV. However, these analyses somewhat experience technical difficulties in defining verifiable criteria for cluster allocations in phylogenetic trees inviting for a more rational approach. Therefore, we applied a relatively new mathematical clustering algorythm named ‘affinity propagation clustering’ (AP) to propose a standardized sub-species classification utilizing full-genome RABV sequences. Because AP has the advantage that it is computationally fast and works for any meaningful measure of similarity between data samples, it has previously been applied successfully in bioinformatics, for analysis of microarray and gene expression data, however, cluster analysis of sequences is still in its infancy. Existing (516) and original (46) full genome RABV sequences were used to demonstrate the application of AP for RABV clustering. On a global scale, AP proposed four clusters, i.e. New World cluster, Arctic/Arctic-like, Cosmopolitan, and Asian as previously assigned by phylogenetic studies. By combining AP with established phylogenetic analyses, it is possible to resolve phylogenetic relationships between verifiably determined clusters and sequences. This workflow will be useful in confirming cluster distributions in a uniform transparent manner, not only for RABV, but also for other comparative sequence analyses.

## Author summary

Rabies is one of the oldest known zoonoses, caused by lyssaviruses. In recent years, more than 21,000 nucleotide sequences for rabies viruses (RABV) have been deposited in public databases. In this study, a novel mathematical approach called affinity propagation (AP) clustering, a highly powerful tool, to verifiably divide full genome RABV sequences into genetic clusters, was used. A panel of existing and novel RABV full genome sequences was used to demonstrate the application of AP for RABV clustering. Using a combination of AP with established phylogenetic analyses is useful in resolving phylogenetic relationships between more objectively determined clusters and sequences. This workflow will help to substantiate a transparent cluster distribution, not only for RABV, but also for other comparative sequence analyses.

## Introduction

Virus taxonomy differs from other types of biological classification because the International Committee on Taxonomy of Viruses (ICTV) not only regulates a Code of Nomenclature, but also considers and approves the creation of novel virus taxa (currently orders, families, subfamilies, genera and species). Thanks to long-lasting efforts of the ICTV [1], the classification of viruses has become clearer and more transparent [2]. Lyssaviruses, negative sense RNA viruses, represent one of 18 currently known virus genera within the family *Rhabdoviridae* of the order *Mononegavirales*. Based on the species concept in virus taxonomy whereby demarcation criteria are established to discriminate between different virus species within a genus [3], the Rhabdovirus study group defines a new species of lyssavirus among other things by more than 18–20% nucleotide divergence within the N-gene compared to the existing lyssavirus species [4]. The Lyssavirus genus comprises 14 recognized and two putative lyssavirus species [2, 5, 6], of which *rabies lyssavirus* represents the prototypical lyssavirus species.

Numerous viral variants of rabies virus (RABV) cause tens of thousands of human deaths annually on a global scale [7]. Nevertheless, there are no further diversification criteria below the species level [2], and for lyssaviruses not even a standard definition for genetic grouping (e.g. lineage, clade, variant, strain, cluster) has been established. In this study, the term ‘cluster’ is utilized throughout the manuscript to define sub-species demarcation.

In order to genetically characterize and sub-classify RABV isolates, multiple studies were conducted, resulting in approximately 21,000 datasets of partial and full genome RABV sequences obtained from the databases of the NCBI. Different studies analyzed the extent of relationship among selected samples using phylogenetic analyses to verify the results. Historically, the majority of phylogenetic analyses have been conducted at regional levels, i.e. for Europe [8, 9], African regions [10, 11], Asia [12] and The Americas [13, 14]. Phylogenetic analyses used mostly N and G gene sequences (Table 1). This is due to a number of factors including the submission of diagnostic RT-PCR amplicon sequences [15]. In fact, the phylogenetic analysis of partial genome sequences requires less computational power and is more cost-effective. Subsequently, even broad-scale phylogenetic analyses are based on partial genes (Table 2)[16]. This approach was further supported by a study indicating that all lyssavirus genes are equivalent for phylogenetic analysis [17]. However, in reality the sequences submitted to NCBI have minimal or no sequence overlaps, resulting in datasets which cannot be compared; furthermore, phylogenetic trees often had low statistical support, as demonstrated previously for Arctic RABVs [18].

**Table 1:** Details of the 46 RABV isolates sequenced in this study.

Genbank Accession numbers	Lab ID	Country of origin	Year of isolation	Species	Taxonomic name	Genome size
LT909545	20282	Afghanistan	2006	dog	*Canis lupus familiaris*	11,929
LT909542	13125	Algeria	1984	dog	*Canis lupus familiaris*	11,931
LT909535	13123	Algeria	1989	dog	*Canis lupus familiaris*	11,928
LT909530	13251	Chile	1979	human		11,931
LT909534	13465	Kenya	1997	jackal	n.d.	11,923
LT909536	13471	Kenya	2001	dog	*Canis lupus familiaris*	11,923
LT909546	13135	Nigeria	1988	cat	*Felis silvestris catus*	11,927
LT909548	13138	Nigeria	1989	dog	*Canis lupus familiaris*	11,923
LT909541	13086	Pakistan	1984	dog	*Canis lupus familiaris*	11,928
LT909531	13177	Sudan	1993	dog	*Canis lupus familiaris*	11,930
LT909528	20520	Tanzania	2009	jackal	n.d.	11,923
LT909551	13473	Ethiopia	1992	dog	*Canis lupus familiaris*	11,927
LT909547	13284	Germany	1990	fox	*Vulpes vulpes*	11,923
LT909537	12951	Estonia	2000	fox	*Vulpes vulpes*	11,923
LT909543	13249	Chile	1973	human	*Canis lupus familiaris*	11,925
LT909538	12989	Finland	1990	fox	*Vulpes vulpes*	11,923
LT909539	13182	India	2002	dog	*Canis lupus familiaris*	11,929
LT909527	13102	Indonesia	1988	dog	*Canis lupus familiaris*	11,930
LT909526	13162	Iran	1991	fox	*Vulpes vulpes*	11,924
LT909550	13020	Norway	2000	fox	*Vulpes lagopus*	11,927
LT909532	12929	Poland	1994	fox	*Vulpes vulpes*	11,924
LT909533	13044	Saudi Arabia	1990	fox	*Vulpes vulpes arabica*	11,924
LT909540	13043	Saudi Arabia	1987	fox	*Vulpes vulpes arabica*	11,924
LT909529	13122	Algeria	1984	dog	*Canis lupus familiaris*	11,928
LT909544	13212	Mexico	2002	dog	*Canis lupus familiaris*	11,925
LT909549	34873	Thailand	1988	unknown		11,930
MG458304	RV50	United States	1975	bat	n.d.	11,922
MG458305	RV108	Chile	unknown	bat	*Desmodus rotundus*	11,923
MG458306	RV860	Czech Republic	unknown	fox	*Vulpes vulpes*	11,924
MG458307	RV995	South Africa	2000	cat	*Felis silvestris catus*	11,922
MG458308	RV1009	South Africa	2000	mongoose	n.d.	11,926
KY860584	RV1124	Turkey	1999	dog	*Canis lupus familiaris*	11,923
MG458309	RV1185	Serbia	1978	dog	*Canis lupus familiaris*	11,923
MG458310	RV1189	Serbia	1986	fox	*Vulpes vulpes*	11,923
MG458311	RV1196	Serbia	1998	fox	*Vulpes vulpes*	11,923
MG458312	RV1219	Serbia	1997	fox	*Vulpes vulpes*	11,923
MG458313	RV1336	Russia	1996	dog	*Canis lupus familiaris*	11,926
MG458314	RV1789	British West Indies	1997	bat	*Desmodus rotundus*	11,922
MG458315	RV2321	Egypt	1998	dog	*Canis lupus familiaris*	11,923
MG458316	RV2322	Egypt	1998	dog	*Canis lupus familiaris*	11,923
MG458317	RV2323	Egypt	1999	dog	*Canis lupus familiaris*	11,923
MG458318	RV2481	South Africa	2008	human		11,918
MG458319	RV2854	Grenada	2011	mongoose	*Herpestes auropunctatus*	11,925
MG458320	RV2924	Nepal	2012	human		11,927
KP723638	RV2985	Ethiopia	2014	wolf	*Canis simensis*	11,926
MG458321	ChDg (RABV)	China	unknown	dog	*Canis lupus familiaris*	11,924

n.d. = not determined. Information on the exact species was not available as at least two different species of jackals, mongoose, and bats are occurring in those particular countries.

**Table 2:** Summary of studies analyzing global RABV sequence diversity.

Target sequence	Number of sequences analyzed	Aim of study	Focus of study	Cluster designation	References
N-gene (220 nt)	61	Epidemiologic and historical evaluations of relationships among RABV isolates	Global analysis of RABV	Numeric and geographic combinations	[50]
N-gene	54	Molecular and phylogenetic analyses to evaluate the intrinsic variability and the evolutionary pattern of RABV N-genes	Global analyses of RABV	Combination of artificial names and numbers (e.g. Vaccine 1) and geographic/ numerical combinations (e.g. Africa1b)	[45]
N-gene (G-gene)	80 (55)	Better understanding of the selection pressures acting on RABV Virus	Global analyses of RABV (focus on selection pressures)	Host associated with geographical belongings (e.g. Skunk (Canada))	[48]
G-L region	65	Determine the population history of the mongoose and canid RABV sequences circulating in Zimbabwe and South Africa	Global analyses (bat RABV as outgroup) focusing on African isolates	Combination of host and geographical origin (e.g. USA skunks, African canids) or regional names (e.g. Middle East)	[51]
N-gene (G-gene)	151 (74)	Stochastic processes of genetic drift and population subdivision are identified as important factors by shaping the global phylogeography of canid RABV	Global analyses of RABV dog related isolates	Geographical names (numerical) (e.g. Asian, Africa-3) Exept: Bat-cluster	[44]
N-gene	228	Provide molecular and virologic evidence that domestic dog rabies is no longer enzootic to the United States and to identify putative relatives of dog-related RVs circulating in other carnivores, we studied	Global analyses of RABV dog related isolates	Geographical, numerical and host combinations	[52]
N-genes of full genomes	22	Elucidate the origin of new RABV isolates circulating in Sri Lanka	Global analyses with study focus on Sri Lanka	(e.g. America, India)	[24]
N-gene (500 nt)	80	Molecular epidemiological study of the Arctic/Arctic-like lineage of RABV to date	(Global analyses without bats) study focus on Arctic regions	Geographical names (lineages & groups) numerical (cluster &subcluster)	[53]
G-genes	172	Investigation of RABV host shifts in the Flagstaff area via large-scale genetic analyses	Global analyses, specific host cluster analyses	Host acronyms or geographical names or acronyms	[25]
Full genomes (and extracted N-genes)	53	Integration of new South Korean isolates into the global RABV distribution	Global analyses, further detailed for Asian isolates	Geographical names	[22]
Full genomes	32	Comparisons of molecular differences between an Isolate from China and one from Mexico, integration of both into global phylogeny	Global analyses, focusing on Asian isolates	Geographical names (numerical: e.g. SEA1)	[23]
Full genomes	36	Evolutional analyses of RABV, quantify the current circulating animal rabies occurrence in Laos and complete the molecular characterization of the viruses	Global analyses focusing on Asian isolates	Geographical names (numerical: e.g. China 1)	[20]
Full genomes	321	Large genome wide evolutionary investigation, aim is to identify those evolutionary patterns and processes associated with host-switching	Global analyses	Geographical names (numerical) (e.g. Asian, Africa-3) Except: Bat-cluster & Rac-SK	[21]
Full genomes	562	Application of APC, a novel mathematical tool for transparent cluster allocation	Global analyses	Geographical names (e.g. Cosmopolitan, New World, Asian, Arctic)	This study

Recent analyses have suggested that full genomes provide better statistical support and are a comprehensive instrument for addressing the evolution, spread and genome-wide heterogeneity of RABV [19]. Increasing sequencing capacities including high-throughput sequencing enable generation of full RABV genome sequences for phylogenetic and evolutionary studies (Table 2). Complete genome analyses provide the ultimate opportunity to detect specific nucleotide substitution patterns and identification of specific motifs across all genes [20]. In addition, full genomes are useful for comparison of selection pressures on different genes and are therefore helpful to analyze cross-species transmission events [20–25], and endemic transmission amongst others [26]. The most comprehensive analysis in this respect investigated the evolutionary history of RABV by contributing 170 newly generated full genome sequences [21].

In all previous phylogenetic studies, cluster allocation was either based on host species, region of origin or statistical (bootstrap) support. However, the allocation of RABVs into clusters can be highly subjective, because the thresholds of statistical support vary depending on the respective dataset, often the sequence length. In combination with the individual designation of clusters at a regional and local level, comparison and combination of published phylogenetic studies on RABV is often difficult. In addition, the increasing number of available RABV sequences represents a challenge for conventional computation of phylogenetic inferences.

Similar problems have been described for other viruses and alternative solutions developed [27–29]. However, to reveal unbiased criteria for cluster definitions we preferred a workflow based on a novel non-hierarchical mathematical clustering method: affinity propagation (AP) clustering in combination with standardized phylogenetic analyses. AP is a tool that was developed for clustering similarity measures between all pairs of input samples based on the concept of "message passing" between data points [30]. The method does not require a vector space structure and so called ‘exemplars’, samples that are most representative for a cluster, are chosen among the observed data samples and not computed as hypothetical averages of cluster samples. These characteristics make AP clustering particularly suitable for applications in bioinformatics [31]. Therefore, this approach has already been successfully applied for various tasks in bioinformatics, e.g. for microarray and gene expression data [30, 32, 33]. Here, an extended panel of existing and newly obtained full genome RABV sequences was used to demonstrate the application of AP for RABV clustering and the results compared with previous studies.

## Methods

### RABV full genome sequences

For AP clustering, a total of 516 RABV full-genome sequences were obtained from the NCBI sequence database using the tools implemented in Geneious ([34], version 10.0.9, http://www.geneious.com). All datasets were manually checked for completeness and the respective missing metadata, e.g. year of isolation, geographical origin, was manually completed where possible from literature (S1 Table).

In addition, a further panel of 46 RABV isolates from previously underrepresented geographical areas such as Near East, Europe, Southern America and some African regions was sequenced using high-throughput sequencing (Table 1). RABVs were obtained from the virus archive of the WHO Collaborating Centre for Rabies Surveillance and Research at the Friedrich-Loeffler-Institut (FLI), Greifswald, Germany, or from the WHO Collaborating Centre for the Characterization of Lyssaviruses, Animal and Plant Health Agency (APHA) Weybridge, United Kingdom. All datasets were subsequently stored in a Geneious (version 10.0.9) database for further use. Due to the high passaging and modifications that are required to produce vaccine strains, these data are not included.

### NGS sequencing

To obtain full genome sequences of 46 isolates, high-throughput sequencing at the two reference laboratories was applied as follows: FLI NGS methods were conducted as described previously [35]. After total RNA extraction from cell culture supernatant DNase (Qiagen, Hilden, Germany) treatment was performed as recommended by the supplier. Briefly, total RNA was extracted from cell culture supernatant using RNeasy Mini Kit (Qiagen) along with on-column DNase (Qiagen) treatment following the supplier recommendations. Subsequently, Agencourt RNAclean XP beads (Beckman Coulter, Fullerton, USA) were used to concentrate and clean the RNA. A maximum of 750 ng RNA was used as input for cDNA synthesis using the cDNA synthesis system kit (Roche, Mannheim, Germany) along with random hexamer primers (Roche). Sequencing libraries were generated using the SPRI-TE instrument with SPRIworks II cartridges (Beckman Coulter) and appropriate adapters as described elsewhere [36]. An Illumina MiSeq platform using a MiSeq reagent kit, version 3 (Illumina, San Diego, USA) was used for sequencing according to the manufacturer's instructions in 2x300 bp paired end mode. The raw reads were quality trimmed and mapped along an appropriate RABV reference using the 454 Sequencing Systems Software suite (version 3.0, Roche). Mapped reads were selected for de-novo assembly (454 Sequencing Systems Software suite) in order to generate full genome RABV sequences.

APHA NGS methods were conducted as described previously [37, 38]. Briefly, RNA was extracted directly from clinical brain material using TRizol, then host genomic DNA and ribosomal RNA was depleted using DNAse (Qiagen) and Terminator 5’-Phosphate-dependent Exonuclease (Epicentre Biotechnologies, Madison, USA) respectively. Preparation of ds cDNA and sequencing libraries is described elsewhere [38]. Sequencing was carried out as above for FLI using 2x150bp paired end mode. An iterative mapping process was implemented as described previously [38] to generate RABV full-genomes.

### Comparative sequence analysis

The sequences were annotated in Geneious ([34], (version 10.0.9, http://www.geneious.com) and submitted to the European Nucleotide Archive (ENA) under study accession number PRJEB22369 (see Table 1 for isolate specific accession numbers). Altogether, 562 full genome RABV sequences were aligned codon-based using ClustalW [39] implemented in MEGA 6 [40]. Subsequently, MEGA 6 was used to conduct phylogenetic analyses, including model test and neighbor joining (NJ) phylogenetic tree calculations [41]. With regard to the model test, the Tamura 3-Parameter (T92) evolution model was applied to all datasets [42]. Both, gamma distribution (five categories) and invariant sites were considered. Statistical tree topology support was derived from 500 bootstrap replications.

### AP clustering

Pairwise distances as calculated in the phylogenetic analysis were merged into a distance matrix and imported to the statistical software R [43]. For further analyses the package “apcluster” was used essentially as described [31]. By default, the AP clustering algorithm determines one sequence among the set of input elements for each potential cluster, which is most representative for this cluster. In AP terminology, these sequences are termed “cluster exemplars” [31]. Since this method was initially developed for analysis of similarity matrices, the distance matrix from the sequence alignment had to be converted by inverting the values. In addition, all values embedded in the matrix were squared to improve robustness and discriminatory power of the analysis. Subsequently, the AP algorithm computes the minimum (pmin) and maximum (pmax) of the input preference (p), which is defined as the tendency of each individual sequence to become a “cluster exemplar”. To define the optimal input preference (p), the number of clusters for the complete preference range (pmin-pmax) was calculated stepwise [31]. Optimal input parameter for intraspecific analyses, i.e. the optimal number of clusters, was defined as the largest range of input parameters for which a constant number of clusters is calculated. This range is termed “plateau” throughout the manuscript. Methodologically, the beginning of the lower bound of the “two” cluster plateau cannot be defined and therefore the length of this plateau was not considered further. According to the now defined optimum input parameter, AP calculated the respective number of clusters and allocated any input sequence to only one of these.

### Mapping of AP clusters

Results of AP were summarized on country level and exported as CSV file into ArcGis Desktop (Esri, version 10.3.1, Redlands, California, USA). Data was visualized in pie charts per country.

### Combination of AP with phylogeny

Prior to phylogenetic analysis, each sequence dataset was completed with the assigned AP cluster using the group allocation function in MEGA 6. Within the phylogenetic tree, a color code for each assigned AP cluster was applied. For better visualization, the tree was condensed to the AP cluster level where applicable, i.e. each condensed branch in the dendrogram exclusively contained sequences unambiguously allocated to one AP cluster.

## Results and discussion

### Phylogenetic analyses of RABV full genomes

All available RABV full genome sequences were used to assess the global evolutionary relationships of RABV. In total, 562 full genome sequences were analyzed, including 46 sequences newly obtained as part of this study. The RABV cohort originated from 76 different countries reflecting a broad host range mainly comprising bats, dogs, raccoons, and foxes (S1 Table). The individual sequence lengths ranged from 11,796 to 11,931 nucleotides in length, representing a lack of terminal sequences which can be problematic to obtain, particularly if amplicon sequence is utilized. Altogether, RABV sequences had a pairwise identity of 86.3% (range: 78.65% - 99.97%) with an average G+C content of 45.3%. The low G+C content (under 50%), indicates an uneven distribution of nucleotides therefore not all evolutionary models are suitable for analysis. Therefore, the T92 model [42] was selected, also indicated by the model test in MEGA 6 [40]. Interestingly, the results described below were equally reproducible with other, more complex and time consuming, evolutionary, models e.g. Generalized Time Reversable (GTR).

Phylogenetic analyses based on neighbor joining (S1 Fig) confirmed previously described cluster distributions (Table 2), whereby two principal RABV clusters were observed. One cluster exclusively contains RABV sequences from The Americas, whereas the other cluster has a worldwide distribution and comprises RABV sequences of terrestrial mammals. In previous studies, the latter was further split into various sub-clusters based on either host or regional allocation, e.g. three [22], five [23], six [44], or up to eleven [45], illustrating methodological differences in the approach of sub-clustering. So far, cluster allocations have only partly been based on metadata including host species and geographic origin (Table 2). Both metadata represent challenges, as host species do not necessarily identify the reservoir host but often the individual spill-over or ‘dead end’ host, whereas the spatial origin of samples is often not detailed enough to enable precise analysis. Additionally, cluster allocation can be by genetic distance within clusters [27] or by statistical (bootstrap) support of resulting nodes in phylogenetic trees [46]. However, delineation of clusters is not defined. In these analyses, 82% (463 out of 564) of all nodes showed bootstrap values higher than 70% (S1 Fig), widely recognized as a threshold for reliability [28], demonstrating that bootstrap support of nodes does not result in the delineation of meaningful clusters, if large numbers of full genome sequences are analyzed [47]. Using genetic distances for cluster allocation alone also does not resolve this issue, as no standards for lyssavirus species are defined for this purpose, and an arbitrarily defined threshold would also be subjective.

### Affinity propagation clustering (AP)

**Fig 1:**
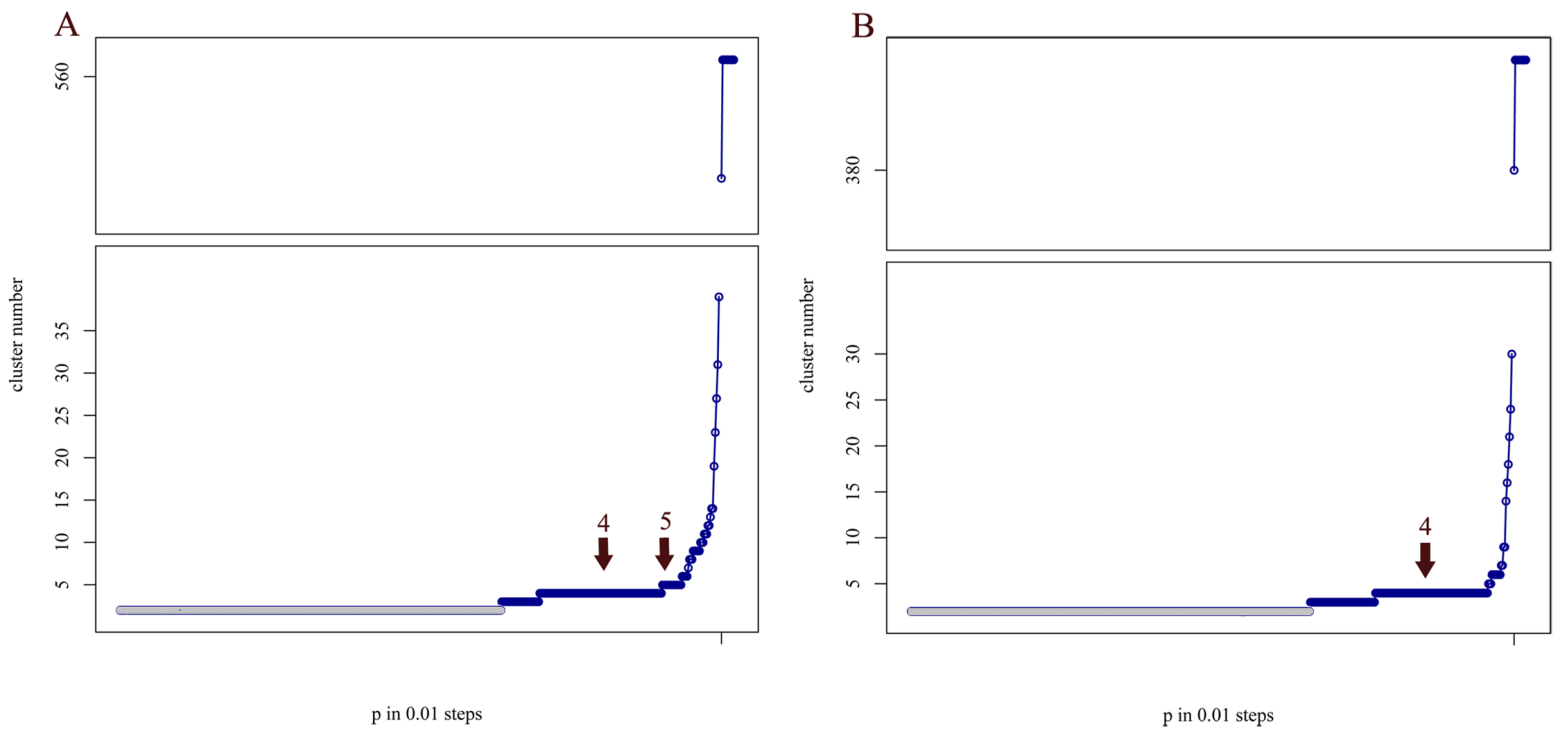
**Graphical display of AP clusters over the range of input parameters for an extended data set (562, A) and a reduced number of sequences (392, B).** Optimal input preference for intraspecific analyses, i.e. the optimal number of clusters, was defined as the largest plateau (here four AP clusters), with the exception of the two cluster plateau (shaded gray) as methodologically, the beginning of the lower bound of the two cluster plateau cannot be defined for certain. In A the increasing length of the five cluster plateaus suggests the existence of an additional AP cluster which is not yet supported by sufficient data.

To address the issue of undefined demarcation within the lyssavirus species, we used AP as a mathematical method for clustering RABV full genome sequences. Besides its computational efficiency [32], which substantially reduces the turnaround time, the main advantage is that it overcomes the described subjective criteria for cluster allocations with the help of mathematical algorithms. The results are non-hierarchically ordered clusters, which can have unequal cluster sizes [30, 32]. By application of AP to pairwise genetic distances from an alignment of all 562 full RABV genomes, the most stable distribution after iteration over all possible input parameters was determined as four clusters (Fig 1A).

**Fig 2:**
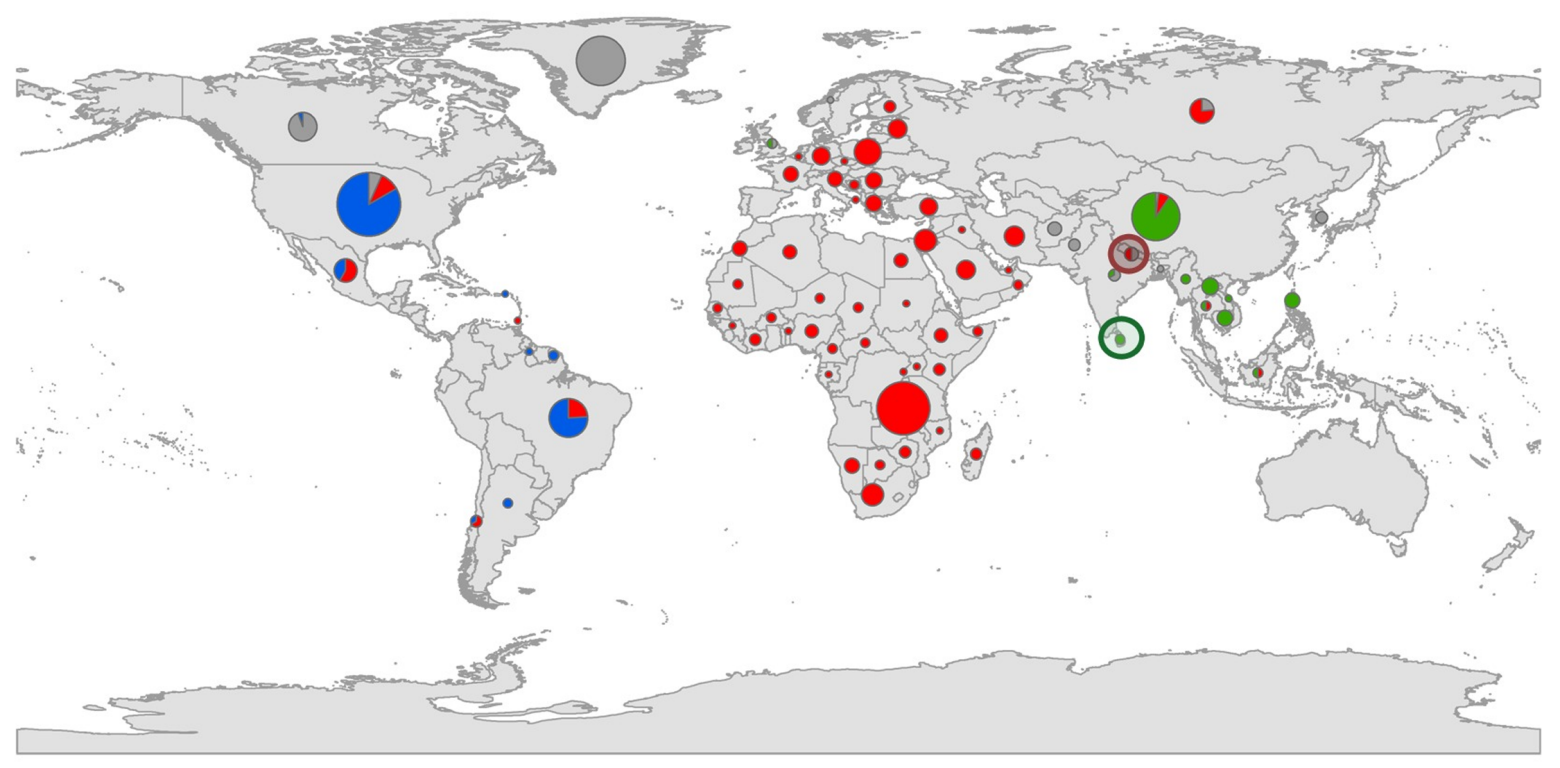
Global distribution of all 562 RABV full-genome sequences according to the results of AP clustering. The width of a pie chart is representing the total number of sequences from a specific country. Forty-six newly generated sequences from previously underrepresented areas in the Near East, Europe, Southern America and Africa were included in this study. The allocation to the AP clusters, i.e. New World cluster (blue), Arctic/Arctic-like (grey), Cosmopolitan (red), and Asian (green) is indicated. The nomenclature of AP clusters was based on previously assigned names. Samples from the previously described Indian subcontinent are highlighted with a red circle (Cosmopolitan sequences), and a green circle (Asian sequences).

These were termed according to previous studies as Arctic/Arctic-like, Cosmopolitan, Asian (together forming the terrestrial cluster) and New World (Table 2). Only one other phylogenetic analysis also identified four main clusters on the basis of full genome sequences [22]. However, other studies identified more than four main clusters for RABV on a global scale [20, 21, 24, 25]. The four clusters identified in our study show a defined spatial distribution (Fig 2), confirming previous analyses that demonstrated the influence of geographic origin rather than the host [48].

In fact, allocation of clusters to known rabies reservoir hosts at this cluster level is problematic. For example, our data indicated that in the Arctic/Arctic like cluster the reservoir host arctic fox is only represented by 59.2% of RABV sequences. In the Cosmopolitan and Asian clusters 44% and 62% of the sequences originate from dog RABVs, respectively, while sequences in the New World cluster were derived to 51.7% from bat RABVs. The remaining sequences include further reservoir species as well as spillover hosts. But sub-dividing these main clusters, may lead to more host-specific clusters.

### Combination of AP and classical phylogeny

**Fig 3:**
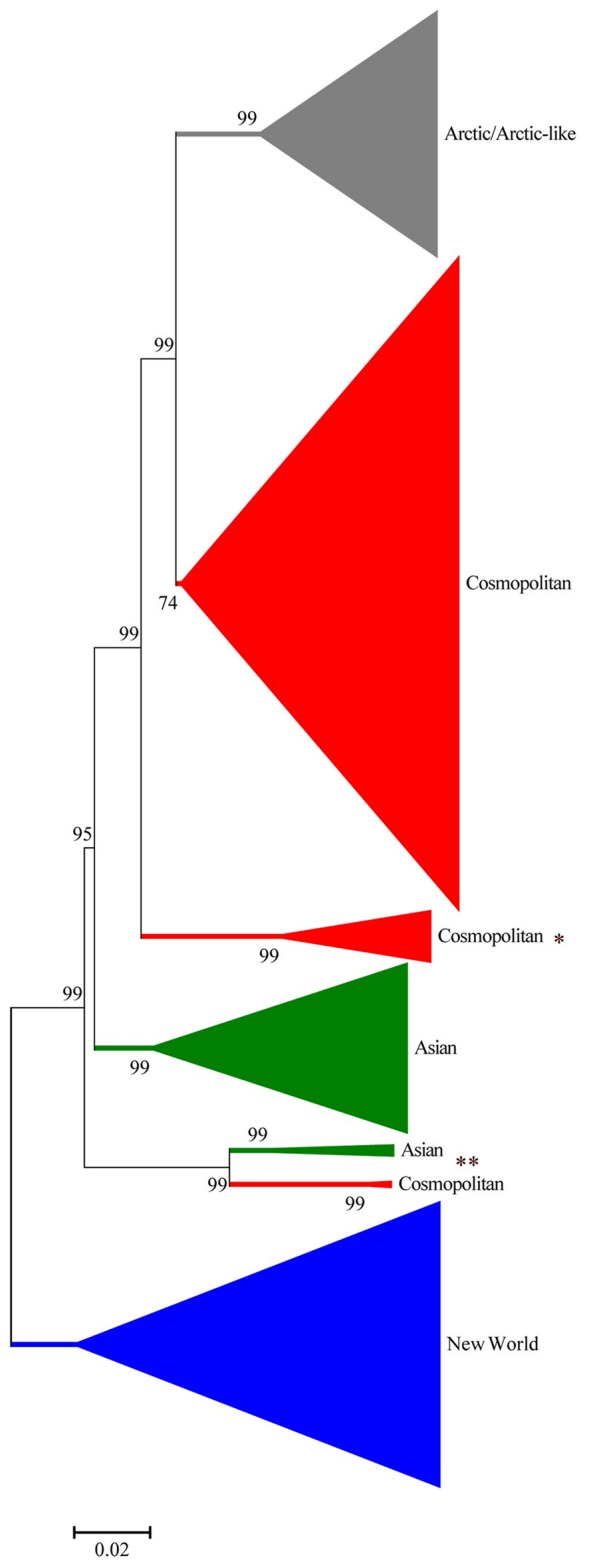
Condensed phylogenetic neighbor joining tree of 562 full genome RABV sequences based on the Tamura-3-parameter evolution model as implemented in Mega 6. Compression is conducted so that a condensed branch only contained sequences of one defined AP cluster. The allocation to the AP clusters, i.e. New World cluster (blue), Arctic/Arctic-like (grey), Cosmopolitan (red), and Asian (green) is indicated. Branches highlighted contain sequences from the Africa-2 lineage (*) and the Indian subcontinent (**).

As AP assigns sequences to robust and reproducable clusters based on transparent input parameter pre-analysis, we tested the hypothesis that a combination of AP with established classical phylogenetic analyses can overcome the inherent limitations of the latter methods alone. Therefore, a comparison of AP and classical phylogeny was undertaken to assess the overall extent of agreement between both methods. The obtained AP cluster distribution was transferred to the calculated phylogenetic dendrogram on an individual sequence basis, and visualized in a compressed tree (Fig 3).

As a result, the phylogenetic tree divides into two main branches. One branch comprises all full-genome sequences allocated to the New World AP cluster (Table 2). The other comprises of sequences from Old World rabies cases, the further branching of sequences in the condensed phylogenetic tree is not congruent with the result obtained by AP except for the Arctic/Arctic-like AP cluster. Sequences allocated to the Asian and Cosmopolitan AP clusters are separated in the dendrogram, whereas sequences of the Asian AP cluster comprise two separate sub-branches. The Cosmopolitan AP cluster sequences are even more diverse (three branches) (Fig 3). These differences are likely a result of the non-hierarchical clustering method of AP in contrast to phylogenetic analysis [32].

The division of RABV sequences into the two closely related branches, Asian and the Cosmopolitan AP cluster (highlighted in Fig 3), seems unclear. These sequences were all RABV variants from the Indian subcontinent, i.e. Nepal, India and Sri Lanka, which should form one joint phylogenetic cluster as suggested previously [21]. In contrast, AP allocates the Nepalese RABV isolates to the Cosmopolitan AP cluster and the Sri Lankan and Indian RABV isolates to the Asian AP cluster. To resolve this discrepancy the predefined AP cluster exemplars (New World AP cluster: JQ685974, Cosmopolitan AP cluster: KR906775, Asian AP cluster: KX148265, and Arctic AP cluster: LT598537) were required in the analyses. The resulting similarity matrix indicates that the similarities of the entire Indian subcontinent sequences to both, the Cosmopolitan (84.86% ± 0.23%) and the Asian (84.98% ± 0.17%) AP cluster exemplars are almost equal (Table 3). Moreover, the distances of the Indian subcontinent sequences to those AP clusters are relatively large, but below the distance to both remaining cluster exemplars (New World: max 17.05%, Arctic: max 15.81%). Therefore, the analyses suggest that the degree of similarity/distance is a weak argument for allocation of these sequences into either the Cosmopolitan or Asian AP cluster. Even geographical allocation cannot resolve this problem (Fig 2). According to these data, AP supports four main clusters. However, whether the two branches really form a separate cluster as suggested recently [21] can only be answered by including more full-genome sequences from those regions in future analyses. An advantage of AP clustering is that the dynamic evolution of further verifiable clusters can already be deduced from the input parameter iteration.

**Table 3:** Similarities in % of the four cluster exemplars to the Asian and Cosmopolitan cluster, additionally similarities from sequences from Indian subcontinent (N = 6) to both Asian and Cosmopolitan cluster exemplars.

Exemplars and individual sequences	Exemplar Cosmopolitan	Exemplar Asian	Exemplar New World	Exemplar Arctic
Exemplar Cosmopolitan	100%	85.22%	82.80%	87.74%
Exemplar Asian	85.22%	100%	83.40%	84.50%
Exemplar Arctic	87.74%	84.50%	82.37%	100%
Exemplar New World	82.80%	83.40%	100%	82.37%
KX148108_Nepal_2011	85.15%	84.86%	83.16%	84.86%
KX148245_Nepal_2009	85.02%	84.75%	83.08%	84.78%
KX148246_India_1997	84.97%	85.21%	83.15%	84.37%
AB569299_Sri Lanka_human_2008	84.67%	85.10%	83.06%	84.36%
AB635373_Sri Lanka_cat_2009	84.53%	84.88%	82.95%	84.19%
KF154999_United Kingdom_dog_2008	84.80%	85.09%	83.18%	84.44%

### Dynamics of AP clustering

In AP, the determination of optimal numbers of clusters is a result of the previously defined iteration over all possible input parameters. To analyze the dynamics of AP in response to the number and diversity of sequences, different datasets were analyzed comprising (i) 392 full-genome sequences (Fig 1B) and (ii) a combined dataset including additional 170 isolates from Troupin et al. [21] (Fig 1A, S1 Table). For both datasets, four AP clusters were supported (Fig 1A and 1B). However, when the larger dataset was used, the plateau supported an increase to five AP clusters (Fig 1A). Interestingly, the extra putative cluster represents the Africa-2 cluster previously defined by phylogenetic analysis [9, 21, 44] and indicated in Fig 3.

**Fig 4:**
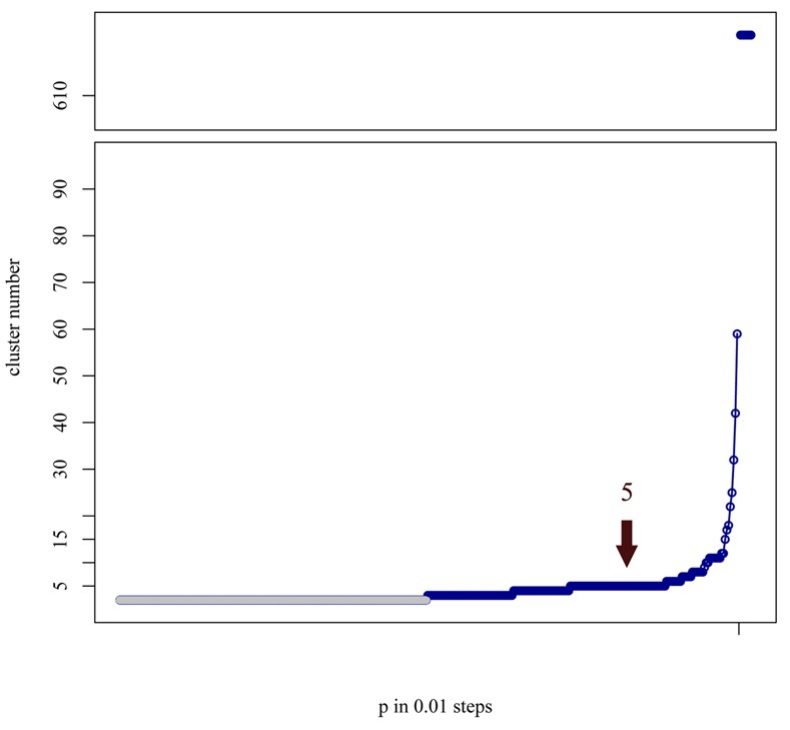
Graphical display of AP clusters over the range of input parameters for G-gene sequences (extracted from full genome sequences, S1 Table) and additional sequences from Nepal (N = 9) and Sri Lanka (N = 49) (S2 Table). G-gene analysis supported the existence of a fifth AP cluster as well as an additionally increased adjacent plateau. As the length of two cluster plateau cannot be defined, it is shaded in gray.

A similar result is observed when partial sequences were analyzed. As mentioned above, the unclear situation for RABV sequences from the Indian subcontinent (Fig 3), i.e. Nepal, India and Sri Lanka, can be resolved by analyzing more sequences from this region. As further full-genome sequences from this area are currently not available for these analyses, an extended panel of G-gene sequences obtained from full genome sequences (S1 Table) and additional isolates from Nepal (N = 11) and Sri Lanka (N = 50) (S2 Table) was evaluated. The analyses further supported the existence of an additional AP clusters (Fig 4). While this additional AP cluster clearly comprises sequences from the Indian subcontinent, the adjacent sixth plateau again was represented by the African-2 phylogenetic cluster. Thus, RABV may be globally separated into five or even more AP clusters if further full genome sequences are available for analysis in the future. The extended analysis of sequences highlights both the dynamics of the AP system and the robustness of cluster allocation. Of course, this is equally true for phylogenetic analysis where the length of sequence and increased geographic coverage of rabies sequences improve the analysis.

### Application of AP for lyssavirus species differentiation

**Fig 5:**
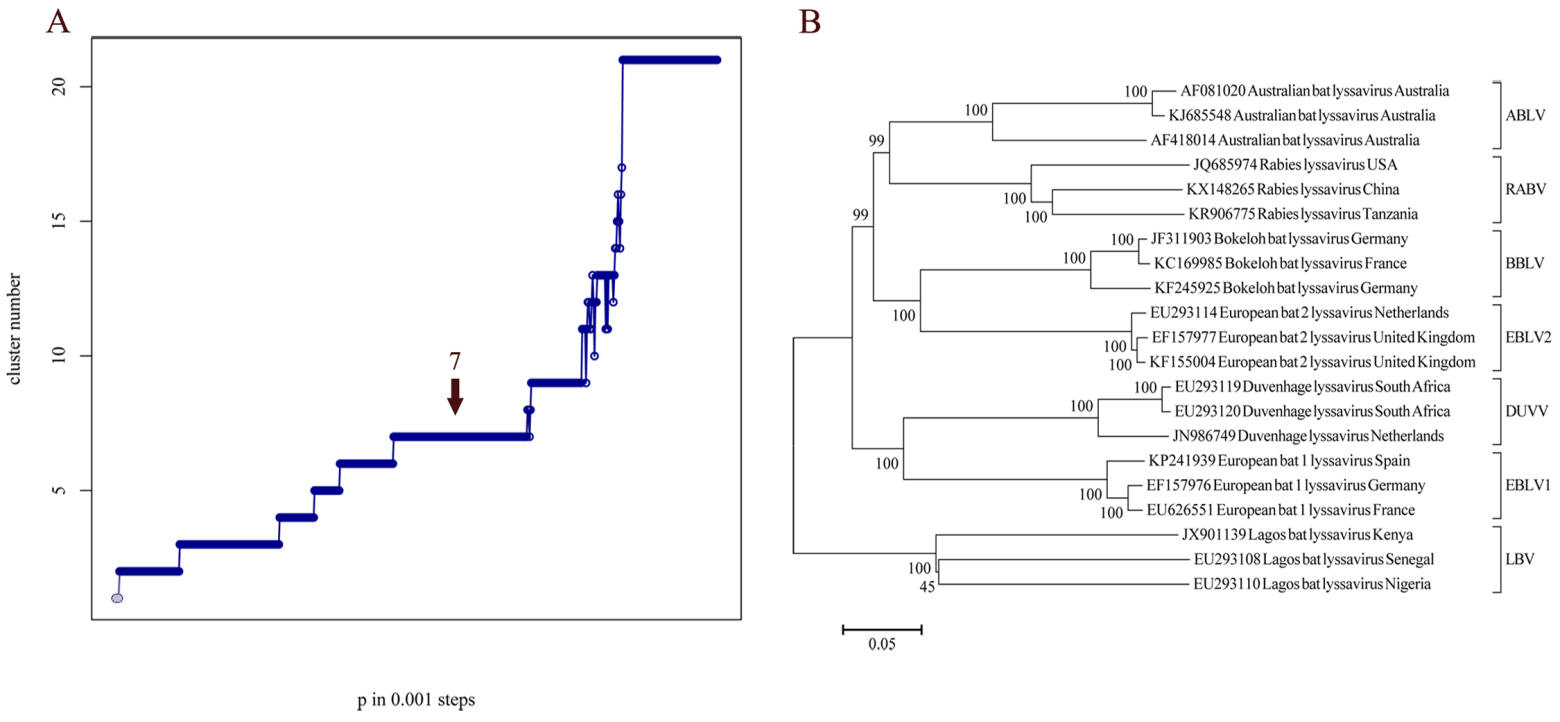
A) Phylogenetic neighbor joining tree of 21 full genome lyssavirus sequences (only lyssavirus species with at least 3 divergent and complete full genome sequences available were used) based on the Tamura-3-parameter evolution model as implemented in Mega6. Bootstrap values (500 iterations) are indicated next to the branches. B) Graphical display of AP clusters over the range of input parameters for the 21 sequences. The largest plateau was seven AP clusters (highlighted). As the length of two cluster plateau cannot be defined, it is shaded in gray.

The lyssaviruses are a rapidly growing genus in the Rhabdoviridae family and we were interested to analyze whether AP is capable of differentiating the lyssavirus species. To this end, a set of 21 representative lyssavirus species full genome sequences were analyzed. The results confirmed the current species delineation and thus could be considered as a species demarcation by ICTV (Fig 5).

Interestingly, for Lagos bat virus (LBV) AP did not segregate the sequences further, although previous studies had suggested that LBV lineage D may represent a separate lyssavirus species [49]. This analysis shows that AP clustering can provide an independent method to resolve questions regarding classification of lyssaviruses into species. However, further full genome sequences from all recognized and putative lyssavirus species would greatly facilitate these analyses.

### Conclusions

The application of AP clustering, phylogenetic analyses, and the combination of both approaches revealed concordant results for RABV sub-species demarcation. The suggested approach offers advantages to phylogenetics in respect to transparency of grouping of RABV isolates and speed. As for the latter, for example the time to calculate AP cluster was about tenfold decreased compared to Neighbour Joining when applying it to the lyssavirus species sequence dataset (Fig 5). Also, the AP workflow simplifies downstream applications, such as mapping of clusters using GIS tools. In addition, we propose a combination of AP with established phylogenetic analyses to resolve phylogenetic relationships between verifiably determined clusters and sequences. This workflow could help to substantiate a transparent cluster distribution, not only for RABV but also for other comparative sequence analyses including virus species delineation as exemplarily shown for lyssaviruses (Fig 5). To this end, the cluster allocation based on AP might be implemented in phylogenetic software packages or sequence analysis pipelines (S2 Fig), and could help to substantiate transparent phylogenetic clustering as suggested previously [47]. In any case, the robustness of sequence analysis is increased by enlarging the dataset preferentially with full genome sequences from previously underrepresented areas accompanied by detailed metadata.

## Supporting information

S1 Table Details of RABV full genome sequences obtained from the National Center for Biotechnology Information (NCBI) database. Datasets used to demonstrate the dynamics of AP in Fig 1A and 1B are highlighted in red.
(XLSX)

S2 Table Details of partial G sequences of RABV from Nepal and Sri Lanka obtained from the National Center for Biotechnology Information (NCBI) database used to resolve the unclear situation for RABV sequences from the Indian subcontinent (Figs 3 and 4). (XLSX)

S1 Fig Phylogenetic neighbor joining tree of 562 full genome RABV sequences based on the Tamura-3-parameter evolution model as implemented in Mega6. Bootstrap values (500 iterations) are indicated next to the branches.
(PDF)

S2 Fig Flow chart showing a suggested pipe-line for analyzing RABV full genome sequences. (TIFF)
