# Supplementary figures and images for: Defining objective clusters for rabies virus sequences using affinity propagation clustering
Source: PLoS Negl Trop Dis. 2018 Jan 22;12(1):e0006182. doi: 10.1371/journal.pntd.0006182 (PMC5794188; doi:10.1371/journal.pntd.0006182)

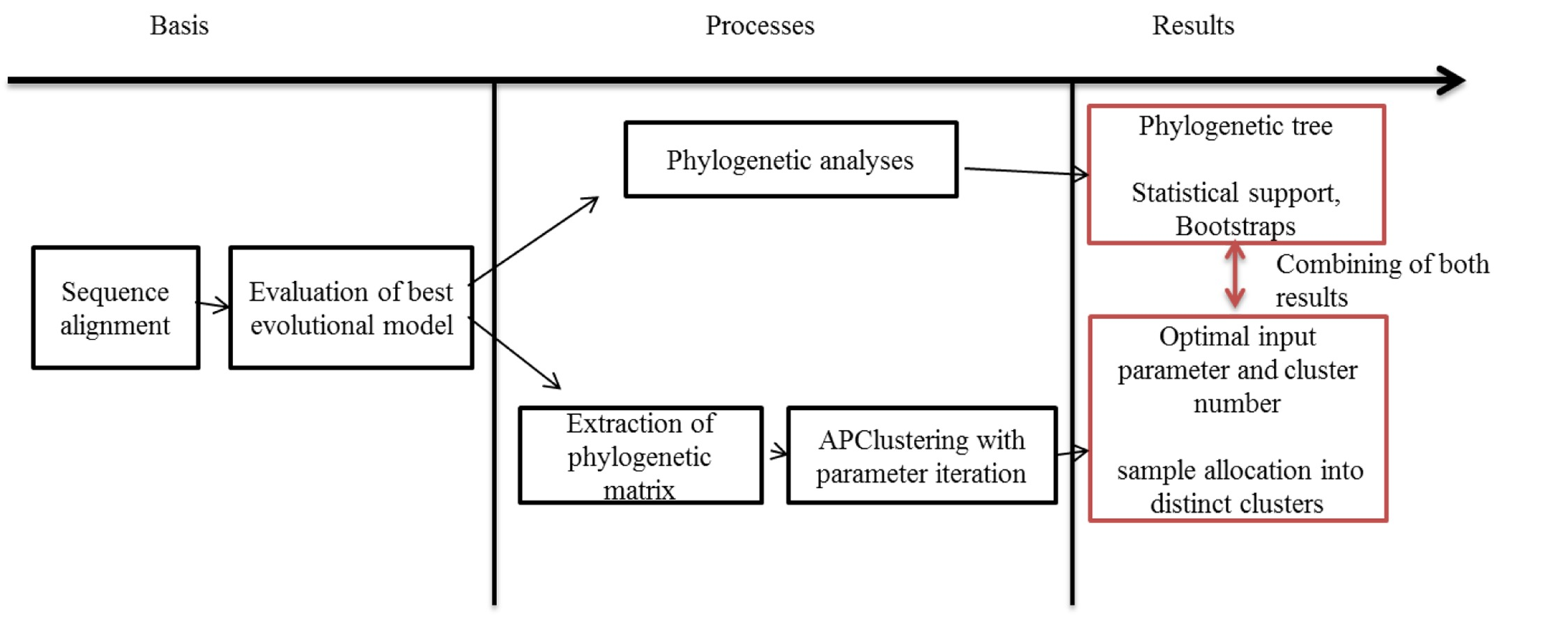

Supplement: S2 Fig — (TIFF) [file pntd.0006182.s004.tiff]
